# DNA data bank of Japan (DDBJ) progress report

**DOI:** 10.1093/nar/gkv1105

**Published:** 2015-11-17

**Authors:** Jun Mashima, Yuichi Kodama, Takehide Kosuge, Takatomo Fujisawa, Toshiaki Katayama, Hideki Nagasaki, Yoshihiro Okuda, Eli Kaminuma, Osamu Ogasawara, Kousaku Okubo, Yasukazu Nakamura, Toshihisa Takagi

**Affiliations:** 1 DDBJ Center, National Institute of Genetics, Shizuoka 411-8540, Japan; 2 Database Center for Life Science, Chiba 277-0871, Japan; 3 National Bioscience Database Center, Japan Science and Technology Agency, Tokyo 102-8666, Japan

## Abstract

The DNA Data Bank of Japan Center (DDBJ Center; http://www.ddbj.nig.ac.jp) maintains and provides public archival, retrieval and analytical services for biological information. The contents of the DDBJ databases are shared with the US National Center for Biotechnology Information (NCBI) and the European Bioinformatics Institute (EBI) within the framework of the International Nucleotide Sequence Database Collaboration (INSDC). Since 2013, the DDBJ Center has been operating the Japanese Genotype-phenotype Archive (JGA) in collaboration with the National Bioscience Database Center (NBDC) in Japan. In addition, the DDBJ Center develops semantic web technologies for data integration and sharing in collaboration with the Database Center for Life Science (DBCLS) in Japan. This paper briefly reports on the activities of the DDBJ Center over the past year including submissions to databases and improvements in our services for data retrieval, analysis, and integration.

## INTRODUCTION

The DNA Data Bank of Japan (DDBJ, http://www.ddbj.nig.ac.jp) (1) is a public database of nucleotide sequences established at the National Institute of Genetics (NIG). Since 1987, DDBJ has been collecting annotated nucleotide sequences, as the traditional DDBJ service, in collaboration with the GenBank (2) at the National Center for Biotechnology Information (NCBI) and the EMBL-Bank (now reorganized as the European Nucleotide Archive, ENA) (3) at the European Bioinformatics Institute (EBI) within the framework of International Nucleotide Sequence Database Collaboration (INSDC) (4). To accept large scale data generated from next-generation sequencing platforms, we, at the DDBJ Center, have launched the DDBJ Sequence Read Archive (DRA), the BioProject for sequencing project metadata, and the BioSample for sample information within the framework of INSDC (5–7). This comprehensive resource of nucleotide sequences and associated information comply with the INSDC policy that guarantees free and unrestricted access to the data archive (8).

Since 2013, the Japanese Genotype–phenotype Archive (JGA, http://trace.ddbj.nig.ac.jp/jga) has been launched in collaboration with our partner institute, the National Bioscience Database Center (NBDC, http://biosciencedbc.jp/en/) of the Japan Science and Technology Agency (1,5). The DDBJ Center provides its database part, which securely stores genotype and phenotype data collected from individuals whose consent agreements authorize data release only for specific research use. JGA allows restricted access to individual data similar to the database of Genotypes and Phenotypes (dbGaP) at NCBI (9) and the European Genome-phenome Archive (EGA) at EBI (10). In collaboration, NBDC provides JGA guidelines and policies for sharing human-derived data (http://humandbs.biosciencedbc.jp/en/guidelines) and reviews data submission and usage requests from researchers.

The DDBJ Center, a division of the NIG, is funded as a supercomputing center. Our services, including web services, submission systems, data retrieval systems, WebAPI, DDBJ Read Annotation Pipeline, and databases are conducted on the NIG supercomputer system. As previously reported (11), the system was replaced by a new commodity-cluster-based system in 2012, and faces the next replacement in 2017.

In this article, we report on submissions and updates to the DDBJ databases during the past year, and introduce our services briefly. In addition, this paper also introduces the active collaboration with the Database Center for Life Science (DBCLS, http://dbcls.rois.ac.jp/en) to develop semantic web technologies for data integration and sharing. We list these achievements independently in the following sections. All resources described here are available from http://www.ddbj.nig.ac.jp and most of the archive data can be downloaded at ftp://ftp.ddbj.nig.ac.jp/.

## THE DDBJ ARCHIVAL DATABASES IN 2015

### Data contents: traditional DDBJ and the DDBJ Sequence Read Archive (DRA)

Between June 2014 and May 2015, the DDBJ periodical release increased by 11 879 389 entries and 31 427 753 923 base pairs. The periodical release does not include whole-genome shotgun (WGS) and third party data (TPA) files (12). The DDBJ has continuously distributed sequence data in published patent applications from the Japan Patent Office (JPO, http://www.jpo.go.jp) and the Korean Intellectual Property Office (KIPO, http://www.kipo.go.kr/en). The JPO transferred its data to the DDBJ directly, whereas the KIPO transferred its data via an arrangement with the Korean Bioinformation Center (KOBIC). The DDBJ contributed 18.39% of the entries and 11.80% of the total base pairs added to the core nucleotide data of INSD. A detailed statistical breakdown of the number of records is shown on the DDBJ homepage (http://www.ddbj.nig.ac.jp/breakdown_stats/prop_ent-e.html). In addition to the above data, the DDBJ has released a total of 10 765 218 WGS entries (769 genomes), 1 182 612 contig/constructed (CON) entries, 773 TPA entries, 6374 TPA-WGS entries, and 1272 TPA-CON entries as of May 29, 2015. In 2014, most nucleotide data submissions to the DDBJ (3882 times; 78.4%) were made by Japanese research groups and the rest came from India (189 times; 3.8%), China (141 times; 2.8%), Thailand (130 times; 2.6%), Iran (111 times; 2.2%), and other countries and regions (501 times; 10.1%).

Notable data sets released from the DDBJ sequence databases are listed in Table 1. Specifically, the DDBJ has released the following: eight cultivars of radish (*Raphanus sativus*) genomes submitted by the Kazusa DNA Research Institute; transcriptome shotgun assemblies (TSA) of intestinal metagenomes of grass carp (*Ctenopharyngodon idella*) submitted by the Institute of Hydrobiology, Chinese Academy of Sciences; expressed sequence tags (EST) of a slime mold (*Acytostelium subglobosum*) submitted by the University of Tsukuba; eggplant (*Solanum melongena*) genome submitted by the Kazusa DNA Research Institute; TSA of *Raphanus sativus* var. *sativus*, *Brassica rapa* subsp. *pekinensis* and their hybrid submitted by the Seoul National University; genome and TSA of two varieties of hops (*Humulus lupulus*) submitted by the Suntory Global Innovation Center Limited; two swallowtail butterflies, common Mormon (*Papilio polytes*) and Asian swallowtail (*Papilio xuthus*), genomes submitted by the University of Tokyo; genomes and transcriptomes of three ants, *Wasmannia auropunctata*, *Monomorium pharaonis*, and *Vollenhovia emeryi* submitted by the Okinawa Institute of Science and Technology; TSA of Welsh onion (*Allium fistulosum*) submitted by the Institute of Vegetables and Tea Science; Antarctic minke whale (*Balaenoptera bonaerensis*) genome submitted by the Kyoto University; genomes of two cultivars of a species closely related to sweet potato (*Ipomoea trifida*) submitted by the Kazusa DNA Research Institute; tadpole shrimp (*Triops cancriformis*) genome submitted by the Keio University; TSA of common iceplant (*Mesembryanthemum crystallinum*) submitted by the Nagoya University; TSA of the Hokkaido salamander (*Hynobius retardatus*), which had been submitted by Hokkaido University; and genomes of STAP-related cell lines submitted by RIKEN.

**Table 1.** List of notable data sets released from the DNA Data Bank of Japan (DDBJ) sequence databases from June 2014 to May 2015

Data type	Organism	Accession numbers for annotated sequences (number of entries)	Accession numbers for raw reads
Genome	Radish (*Raphanus sativus* cv. Aokubi S-h)	WGS: BAUK01000001-BAUK01101710 (101,710 entries)	DRR014095-DRR014097
		scaffold CON: DF384214-DF396802 (12,589 entries)	
	Radish (*Raphanus sativus* cv. Sayatori)	n/a	DRR014098
	Radish (*Raphanus sativus* cv. Taibyosobutori)	n/a	DRR015470
	Radish (*Raphanus sativus* cv. Yumehomare)	n/a	DRR015471
	Radish (*Raphanus sativus* cv. Sakurajima)	n/a	DRR015472
	Radish (*Raphanus sativus* cv. AZ26H)	n/a	DRR015473
	Radish (*Raphanus sativus* cv. N1–3)	n/a	DRR015474
	Radish (*Raphanus sativus* cv. Nishimachi-Risou)	n/a	DRR015475
	Eggplant (*Solanum melongena*)	WGS: BAUE01000001-BAUE01143048 (143,048 entries)	DRR014074-DRR014076
		scaffold CON: DF357214-DF384212 (26,999 entries)	
	Hop (*Humulus lupulus* var. *cordifolius*)	WGS: BBPB01000001-BBPB01292698 (292,698 entries)	DRR024452-DRR024456
		scaffold CON: LD000001-LD132476 (132,476 entries)	
	Hop (*Humulus lupulus* var. *lupulus*)	WGS: BBPC01000001-BBPC01292698 (292,698 entries)	DRR024392-DRR024447
		scaffold CON: LD132477-LD264952 (132,476 entries)	
	Common Mormon (*Papilio polytes*)	WGS: BBJD01000001-BBJD01014374 (14,374 entries)	n/a
		scaffold CON: DF820621-DF824493 (3,873 entries)	
	Asian swallowtail (*Papilio xuthus*)	WGS: BBJE01000001-BBJE01010777 (10,777 entries)	n/a
		scaffold CON: DF824494-DF830065 (5,572 entries)	
	Little fire ant (*Wasmannia auropunctata*)	WGS: BBSV01000001-BBSV01103610 (103,610 entries)	DRR029036-DRR029065
		scaffold CON: LD264953-LD342581 (77,629 entries)	DRR031468-DRR031483
	Pharaoh ant (*Monomorium pharaonis*)	WGS: BBSX01000001-BBSX01042927 (42,927 entries)	DRR023370
		WGS: BBSX02000001-BBSX02024607 (24,607 entries)	
		scaffold CON: DF954455-DF967519 (13,065 entries)	
	*Vollenhovia emeryi*	WGS: BBUO01000001-BBUO01023916 (23,916 entries)	DRR030167-DRR030174
		scaffold CON: DF939120-DF952377 (13,258 entries)	DRR030402
	Antarctic minke whale (*Balaenoptera bonaerensis*)	WGS: BAUQ01000001-BAUQ01720900 (720,900 entries)	DRR014695
		scaffold CON: DF397027-DF818470 (421,444 entries)	
	Marbled flounder (*Pseudopleuronectes yokohamae*)	WGS: BBOV01000001-BBOV01525502 (525,502 entries)	DRR024206
	A species closely related to sweet potato	WGS: BBOG01000001-BBOG01163047 (163,047 entries)	DRR023905-DRR023907
	(*Ipomoea trifida* cv. Mx23Hm)	scaffold CON: DF850533-DF884990 (34,458 entries)	
	A species closely related to sweet potato	WGS: BBOH01000001-BBOH01377770 (377,770 entries)	DRR023898-DRR023904
	(*Ipomoea trifida* cv. 0431–1)	scaffold CON: DF884991-DF933566 (48,576 entries)	
	Tadpole shrimp (*Triops cancriformis*)	WGS: BAYF01000001-BAYF01060629 (60,629 entries)	DRR017999
	STAP-related mouse cell lines (*Mus musculus*)	n/a	DRR028632-DRR028661
EST	Slime mold (*Acytostelium subglobosum*)	5′-EST vegetative stage: HY448297-HY457975 (9,679 entries)	n/a
		3′-EST vegetative stage: HY457976-HY467594 (9,619 entries)	
		5′-EST vegetative stage: HY467595-HY470588 (2,994 entries)	
		3′-EST vegetative stage: HY470589-HY473646 (3,058 entries)	
		5′-EST developmental stage: HY473647-HY491366 (17,720 entries)	
		3′-EST developmental stage: HY491367-HY508708 (17,342 entries)	
TSA	Fish metagenome (intestinal contents of *Ctenopharyngodon idella*)	LA000001-LA022570 (22,570 entries)	DRR013890
		LA022571-LA050083 (27,513 entries)	DRR013891
		LA050084-LA080192 (30,109 entries)	DRR013892
		LA080193-LA098774 (18,582 entries)	DRR013893
		LA098775-LA118857 (20,083 entries)	DRR013894
		LA118858-LA151643 (32,786 entries)	DRR013895
		LA151644-LA221552 (69,909 entries)	DRR013896
		LA221553-LA244919 (23,367 entries)	DRR013897
		LA244920-LA284088 (39,169 entries)	DRR013898
		LA284089-LA296882 (12,794 entries)	DRR013899
		LA296883-LA328022 (31,140 entries)	DRR013900
	*Raphanus sativus var. sativus*	FX657517-FX691702 (34,186 entries)	DRR014232, DRR014233
	*Brassica rapa subsp. pekinensis*	FX691703-FX725989 (34,287 entries)	DRR014230, DRR014231
	Hybrid (*Brassica rapa x Raphanus sativus*)	FX725990-FX799344 (73,355 entries)	DRR014228, DRR014229
	Hop (*Humulus lupulus var. lupulus*)	LA328023-LA715951 (387,929 entries)	DRR024457-DRR024463
	common iceplant (*Mesembryanthemum crystallinum*)	FX891461-FX944976 (53,516 entries)	DRR018522-DRR018525
	Hokkaido salamander (*Hynobius retardatus*)	LE000001-LE740933 (740,933 entries)	DRR016729-DRR016800
	Pharaoh ant (*Monomorium pharaonis*)	LA777684-LA901618 (123,935 entries)	DRR023701-DRR023724
			DRR029611-DRR029634
			DRR032044-DRR032266
Transcriptome	Little fire ant (*Wasmannia auropunctata*)	n/a	DRR029066-DRR029098
	*Vollenhovia emeryi*	n/a	DRR030152-DRR030166

### The Japanese genotype-phenotype archive (JGA)

As of 1 September 2015, JGA has archived 33 studies (8.1 TB) of individual-level human data submitted by Japanese researchers. Archived studies include exome sequence analysis of cancer and other diseases, epigenetic analysis of Hepatitis B virus integration site, and magnetic resonance imaging of brain from bipolar disorder individuals. Submission of these studies has been reviewed and approved by the Data Access Committee (DAC) at NBDC. The summaries of 19 studies are public both on the JGA (https://ddbj.nig.ac.jp/jga/viewer/view/studies) and NBDC (http://humandbs.biosciencedbc.jp/en/data-use/all-researches) websites. To access individual-level data of these public studies, users need to apply data access requests to the NBDC (http://humandbs.biosciencedbc.jp/en/data-use).

## DDBJ SYSTEM PROGRESS

### Update registration systems for the DDBJ traditional assembled sequence archives

For data submission to the traditional DDBJ database, we provide two systems: the Nucleotide Sequence Submission System (NSSS; 5) and the Mass Submission System (MSS; 13). NSSS is an interactive application to enter all items via a web-based form; http://www.ddbj.nig.ac.jp/sub/websub-e.html. The MSS is a procedure to send large-scale data files directly; http://www.ddbj.nig.ac.jp/sub/mss_flow-e.html. Both systems were enhanced to apply the new rules of feature and qualifier usages (see http://www.ddbj.nig.ac.jp/insdc/icm2014-e.html#ft). Previously, validation tools for MSS were built with Java 7. In February 2015, the tools were re-built with the new version, Java 8, because of the end of public updates for Java 7 after April 2015.

### New functions for DRA/BioProject/BioSample submission systems

**Figure 1.**
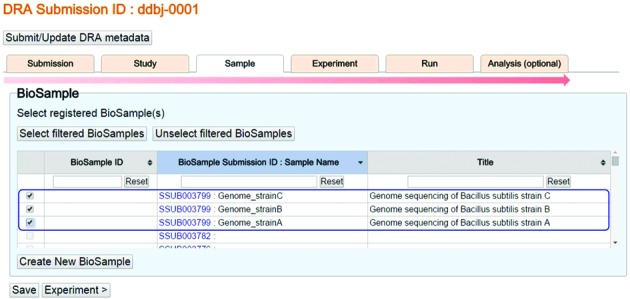
New BioProject/BioSample/DRA submission interface. The new BioProject, BioSample and DRA submission interface enables submission of DRA metadata objects (Submission, Experiment, Run and Analysis) referencing submitted but yet un-accessioned BioProject and BioSample objects. Users can submit new BioProject, BioSample, and DRA metadata at the same time.

In April 2015, we released the enhanced BioProject/BioSample/DRA submission system. This system enables the users to submit a DRA submission referencing submitted but yet un-accessioned BioProjects and BioSample objects; thus, they need not wait for BioProject and BioSample accession numbers before submitting sequencing data to DRA (Figure 1).

### Sequence analytical services

#### The NIG supercomputer as a sequence analytical platform

The NIG supercomputer consists of calculation nodes for general-purpose (554 thin-nodes, each with 64 GB of memory) and memory-intensive tasks including *de novo* assembly of sequencing reads (10 medium nodes each with 2 TB of memory and 1 fat node with 10 TB of memory). These nodes are interconnected with InfiniBand QDR/FDR by a complete bisection fat-tree topology. For the massive data analysis, the NIG supercomputer is equipped with 7 PB of the Lustre parallel distributed file system (http://lustre.org), and for archiving of the Sequence Read Archive data, the 5.5 PB MAID system (http://sc.ddbj.nig.ac.jp/index.php/en/en-sysconfig2/en-hardconfig) (1,11). The number of NIG Supercomputer users increased from 1384 at 1 June 2014 to 2016 at 31 May 2015.

#### Supported analytical tools and public datasets in the NIG Supercomputer

NIG operates the supercomputer facilities for the purpose of (i) construction and archiving the DDBJ databases, and providing analysis services on them (ii) making research and educational resources available to life science researchers in Japan. For the convenience of the login users, many popular tools and libraries in the bioinformatics domain were installed in the system, as shown on the home page (http://sc.ddbj.nig.ac.jp/index.php/ja-avail-oss).

In order to help reproduce previously executed analysis flow, different versions of the analytical tools are installed in different search paths. Pre-installed datasets in the NIG supercomputer for those analytical tools are listed on the webpage (http://sc.ddbj.nig.ac.jp/index.php/ja-availavle-dbs).

#### WebBLAST, ClustalW, VecScreen, ARSA and WebAPI

The DDBJ Center provides Web BLAST (14), ClustalW (15,16), and VecScreen (http://www.ncbi.nlm.nih.gov/tools/vecscreen/univec) services, which receive requests from web interfaces. The DDBJ Center also provides the new version of Web API for Bioinformatics (WABI) (17–19), the RESTful Web API service that can process requests from computer programs. The WABI service includes BLAST, VecScreen, ClustalW, MAFFT (20,21), getentry data retrieval system via accession numbers, and the ARSA keyword search system for the DDBJ flat files (11).

#### TXSearch to retrieve NCBI taxonomy index

**Figure 2.**
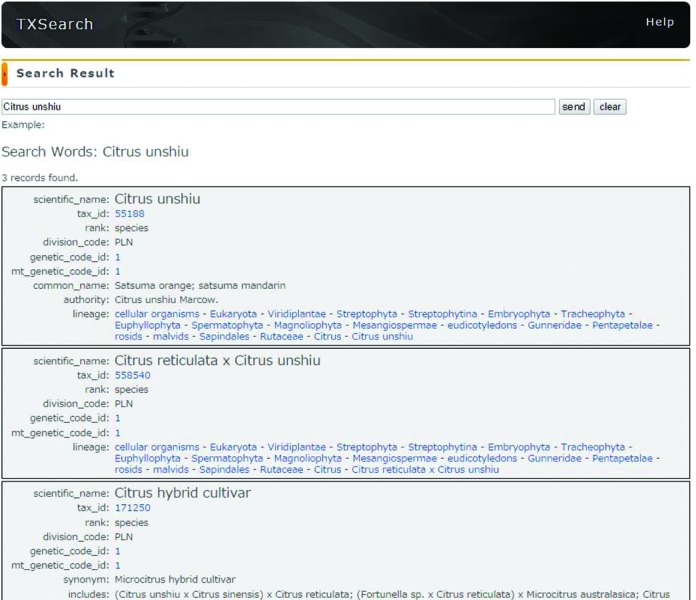
Screenshot of the DNA Data Bank of Japan (DDBJ) TXSearch tool

TXSearch (http://ddbj.nig.ac.jp/tx_search/) is an NCBI Taxonomy browsing system in the DDBJ. This browsing system allows data submitters to find authentic scientific names used in the INSDC for the purpose of vocabulary control. Due to the replacement of the NIG supercomputer in 2012, we re-implemented most of our services on open source middleware to become accommodated to the new system. The TXSearch system was built on the Apache Solr full text search system and MySQL. The RESTful Web API service is also provided as shown in Figure 2. The data in the TXSearch are updated in daily bases by downloading the NCBI Taxonomy database (22) from the NCBI FTP site (ftp://ftp.ncbi.nih.gov/pub/taxonomy).

#### A virtual machine image for the DDBJ Pipeline

The DDBJ Read Annotation Pipeline (DDBJ Pipeline, http://p.ddbj.nig.ac.jp) is a high-throughput web annotation system of next-generation sequencing reads running on the NIG supercomputer (23). The pipeline's basic component is for reference genome mapping and *de novo* assembly, and subsequent analysis such as structural and functional annotations with a Galaxy interface (24). In 2015, a virtual machine image was generated for the purpose of providing operations in a non-NIG supercomputer environment, but under other cloud computer environments. Researchers may utilize the virtual machine image for users’ sensitive datasets such as human personal genomic sequences.

#### INSDC ontology and BioSample attribute RDF

To improve the reusability of the sequence annotation data, we have developed a system to make the DDBJ records into the Resource Description Framework (RDF) version in collaboration with DBCLS (25,26). We applied the system to produce RDF triple datasets of the entire DDBJ records based on the INSDC ontology, which describes semantics of the INSDC sequence records and the FALDO ontology to annotate locations of sequence features. We enhanced the INSDC ontology to be applied to DDBJ submission systems such as D-easy, DRA, BioSample, BioProject under collaboration with the RIKEN BioResource Center (BRC) Institute. To semantically integrate, we constructed a dataset for BioSample attributes RDF in BioHackathon 2014 (http://2014.biohackathon.org/).

## FUTURE DIRECTION

In this report, we introduced updates of the DDBJ data sets, data submissions, and analytical systems during the past year. We plan to develop a unified submission portal website for all database systems, in concert with the replacement of our supercomputing system in every 5 years (the next replacement year is 2017). Especially, the JGA system needs update to efficiently archive and distribute ever-growing volume of human genome sequencing data. In terms of RDF, application software is under development as the Microbial BioSample OWL. The current foci on future enhancements of the computer infrastructure in DDBJ are (i) refinement of management process and security infrastructure for JGA; (ii) provision of a computing infrastructure suitable for developers and data analysts on HPC environment; and (iii) performance enhancement of data processing for INSDC database construction and usability. For HPC developers, we are constructing an experimental system for OpenStack private cloud environment on the NIG supercomputer, in addition to the extension of Docker systems for DDBJ analytical services.
